# Growth, Morphological, and Physiological Responses to Drought Stress in *Bothriochloa ischaemum*

**DOI:** 10.3389/fpls.2017.00230

**Published:** 2017-02-24

**Authors:** Ying Liu, Peng Li, Guo Ce Xu, Lie Xiao, Zong Ping Ren, Zhan Bin Li

**Affiliations:** ^1^Institute of Water Resources and Hydro–electric Engineering, Xi'an University of TechnologyXi'an, China; ^2^State Key Laboratory Base of Eco-Hydraulic Engineering in Arid Area, Xi'an University of TechnologyXi'an, China

**Keywords:** *Bothriochloa ischaemum*, stable carbon isotope, root morphology, water use efficiency, drought stress

## Abstract

Water shortage in the arid-semiarid regions of China seriously hampers ecosystem construction. Therefore, elucidation of the mechanisms by which vegetation in that area responds to drought stress may enable us to improve utilization of limited water resources and thus contend with the problem of drought and water shortage. We studied *Bothriochloa ischaemum*, a native grass species, conducted potting control tests to compare several indicators of *B. ischaemum* grown under three different moisture conditions (80%, 60%, 40% Field capacity represent sufficient water supply, mild water stress, and serious water stress, respectively). Plant response parameters measured included biomass accumulation, root morphology, transient water use efficiency (WUE), stable carbon isotope ratio (δ^13^C), and stable carbon isotope discrimination (Δ^13^C) of various plant organs and their interrelationships. *B. ischaemum* had the greatest WUE under mild drought stress. However, serious drought stress resulted in considerable decline in overall biomass but substantial increase in root-to-shoot ratio and fine-root biomass. Coarse-root biomass dropped appreciably, indicating that serious drought stress leads to allocation non-uniformity of the carbon “sink.” δ^13^C and Δ^13^C of stem correlated considerably with root morphology, suggesting the feasibility of characterizing WUE, biomass, and root morphology of *B. ischaemum* via the stable carbon isotope approach. Our evaluation of 21 drought resistance indicators of *B. ischaemum* showed that under a given moisture treatment gradient one can isolate an optimal indicator to express growth, morphology, and physiology, to improve the accuracy of depicting plant drought resistance and simplify the drought resistance indicator system. This study elucidates the response mechanism of *B. ischaemum* to drought stress and provides theoretical support to screening of drought-resistant plants across the arid-semiarid regions of China.

## Introduction

Water resources are scarce in arid-semiarid regions of China, which is not only the main limiting factor of ecological system in this area, but also the key factor to control ecosystem structure (Zou et al., 2016). Rainfall is a major means of recharging the vegetation ecosystem (Shan, 2002), but scant precipitation and non-uniform annual and quarterly allocation often become the limiting factors of local plant growth and vegetation restoration (Xu et al., 1997; Wei et al., 2004). Since the Chinese government implemented the “Grain for Green” policy in 1999, cropland area in western China has decreased dramatically while vegetation area has increased (Lu et al., 2012); this transformation has led to increased public focus on vegetation utilization of limited soil water resources. Related studies show that, plants can only absorb and utilize a small share of soil moisture (Huang et al., 2004; Wang et al., 2006), these studies suggest that identifying the anti-drought mechanism(s) of local plants can address the water shortage in this area and thus further improve crop utilization of the soil water resource.

Plant water-use efficiency (WUE) reflects water consumption and drought adaptability of a plant (Martin et al., 1999; Ray et al., 1999); high WUE is a mechanism of plant adaptation to water deficit and an important characteristic of plant response to an arid environment (Jaleel et al., 2008; Sun et al., 2008). Moreover, several studies indicate that plant root systems play an important role in plant adaptation to a drought stress habitat (Toorchi et al., 2002; Pu et al., 2010; Zhang et al., 2012). Traditional wisdom holds that a plant subjected to drought stress in the Loess Plateau area would show constant or increased WUE (Xu et al., 2003, 2010), increased root system biomass, and a morphology approaching deep and dense roots (Passioura, 1983; O'Toole and Bland, 1987; Blum and Sullivan, 1997). Nonetheless, other scholars have argued that if the root system is too large, the WUE would instead decline (Mu et al., 2005). Therefore, conflicting opinions suggest the need for further study of the adaptation strategy of plant morphology and physiology to drought in the arid-semiarid regions of China.

Recent studies of WUE for the Qinghai-Tibetan Plateau, Northeast China grassland, and tropical and subtropical plants in China have utilized stable carbon isotope analysis. These studies demonstrate that stable carbon isotope discrimination (Δ^13^C) and stable carbon isotope ratio (δ^13^C) of plant leaves correlate strongly with transient or prolonged WUE and can indicate plant adaptation to drought (Su et al., 2000; Qu et al., 2001; Chen et al., 2013; Zhou et al., 2013; Qiu et al., 2014). The related studies also mention that stable carbon isotope fractionation occurred to different organs of C_3_ and C_4_ plants (Zhao et al., 2004; Zhang et al., 2009; Ghashghaie et al., 2016), and the role of stable carbon isotope characteristics of each organ in defining plant WUE was supported to some extent. However, studies have not yet been reported on the possibility that stable carbon isotope characteristics can characterize root system anti-drought properties. Summarizing the studies of other researchers, we proposed to test the hypothesis that, compared with all other organs of a plant, one organ of the plant has a stable carbon isotope eigenvalue more relevant to WUE, biomass, and root system morphological properties; stable carbon isotope characteristics of this organ should allow us to characterize plant WUE, growth, and root system morphological features.

The above studies also demonstrate that plant drought resistance is an overall reflection of multiple factors; therefore, to understand plant drought resistance, it is important to reveal the anti-drought mechanisms of herbaceous plants in the arid Loess Plateau area by analyzing differences in given plant growth indicators (e.g., biomass allocation to the aboveground and underground portions), morphological indicators (e.g., root system morphology), and physiological indicators (WUE, Δ^13^C, and δ^13^C). However, to our knowledge there are few studies that utilize the stable carbon isotope technique to comprehensively evaluate and screen drought resistance indicators of herbaceous plants in the arid-semiarid regions of China. Therefore, we put forward the hypothesis: among plant growth, morphological, and physiological indicators, there is a best indicator which is more relevant to drought resistance, and its use can reflect plant drought resistance more concisely and accurately.

*Bothriochloa ischaemum*, a major grass species for creating grassland in the arid-semiarid regions farmland-to-grassland conversion project, is also an important species for restoration and carbon storage of degraded grassland (Xu et al., 2007; Cheng et al., 2010). Characterized by its superior regenerative capacity, quick reproduction, drought and trampling resistance, notable regional adaptability, and high productivity, it constitutes a high-quality natural forage in the arid-semiarid regions of China. Its root system is well developed and forms a network that can intercept rainfall and conserve soil and water. We carried out moisture-controlled potting tests to study biomass accumulation and allocation of *B. ischaemum* under different soil moisture conditions. Our observations included root system morphological characteristics and the relationship of transient WUE vs. δ^13^C and Δ^13^C values of natural abundance of each organ, so as to elucidate the response mechanism of *B. ischaemum* to drought stress and provide theoretical support to construction and management of man-made grassland in the arid-semiarid regions.

## Materials and methods

### Experimental materials and design

The experiment was carried out at Xi'an University of Technology in June 2014 using self-made transparent cuboid Plexiglas containers (19 × 4 × 27 cm). The test soil was collected from the *B. ischaemum*-dominated grassland at Wangmao gully in Suide, North Shaanxi; after superficial humus and litter were cleared away, a 0- to 30-cm layer of soil was removed, mixed well, bagged, and air-dried naturally. Thereafter, the soil was passed through a 2-mm sieve and its bulk density measured. Each Plexiglas pot was packed with 2.5 kg of soil, and the soil bulk density was 1.2 g cm^−3^, identical to that of the soil sampling plot in North Shaanxi. The test soil total nitrogen was 0.69 g kg^−1^ and its field capacity (FC) was 22%. The soil is classified as Calcic Cambisol (FAO-UNESCO, 1977) and is mainly a wind-deposited loess soil that is highly erosive.

Seeds of *B. ischaemum* for the test were harvested in October 2013 from natural grassland in Ansai, North Shaanxi; harvested seeds were stored in paper bags under natural conditions in the laboratory. On 01-May-2014 five bunches of seeds were sown by scattering in each container; the individual seedling growing most vigorously in each bunch was retained after seedling emergence. Seed holes lacking seedling emergence were replanted. The same seeds which for replanting were sown in the same soil conditions at the same time during the experiment. After seedling thinning, moisture control treatment was begun on 01-Jul-2014, using three soil moisture gradients: sufficient water supply (80% FC as control, CK), mild stress (60% FC, mild water stress, MS), and serious stress (40% FC, serious water stress, SS); nine replicate pots were prepared for each moisture gradient, for a total of 27 pots of samples. In the absence of stable carbon, hydrogen, and oxygen isotope labeling, stable carbon isotope ratios of all test samples were of natural abundance.

Potting soil moisture was controlled by means of weighing: first of all, the pots with the biomass of *B. ischaemum* was weighted and recorded of all the moisture control treatment as the background weight. The background weight of CK, WS and SS is 3,875, 3,765, and 3,655 g/pot. Then the pots were weighed at 18:00 every day, and watered if the weight was lower than the background weight until the experiment ended on 11-Nov-2014. The biomass of each pot of *B. ischaemum* was included in the background weight before the moisture control treatment. Because the difference between the initial biomass of seedling (0.867 g) and the maximum wet biomass of each pot of *B. ischaemum* (5.812 g) following the test was only 0.1% of the total weight of the overall moisture-controlled pot (3880 g), and the moisture control duration is short, the effects of *B. ischaemum* growth weight gain on moisture treatment control were neglected.

### Experimental methods and determinations

#### Determination of photosynthetic characteristics and transient WUE of *B. ischaemum*

The experiment was carried out during the period from 09:00 am to 11:00 am on 03-Aug-2014, 10-Aug-2014, and 13-Aug-2014, when the weather was sunny and cloudless (during the early-flowering phase of the *B. ischaemum* growing period). The 2- by 3-cm transparent leaf chamber of an Li-6400 portable photosynthesis system (LI-COR, Inc., Lincoln, NE) was used to determine photosynthetic characteristic indicators of *B. ischaemum* and environmental parameters under natural conditions (natural light source; uncontrolled CO_2_ concentration and humidity): net photosynthetic rate of leaf (*Pn*), transpiration rate (*Tr*), stomatal conductance (*Gs*), and intercellular CO_2_ concentration (*C*_*i*_). Sufficiently spread leaves were selected for determination, and every point was stabilized for 2 min before reading. Based on the above determination parameters, leaf WUE was calculated as follows: WUE = *Pn*/*Tr*.

#### Determination and calculation of root system morphological indicators and seedling biomass of *B. ischaemum*

On 11-Nov-2014 the aboveground portion of all *B. ischaemum* plants of each pot was mowed, numbered, and stored separately as new leaves, old leaves, and stems. The soil in each pot along with the root system was poured onto a plastic cloth, and the root system was immediately hand-picked from the soil while distinguishing between the taproot (coarse root) and the lateral roots (fine roots), separately (the taproots were kept separate from lateral roots), then placed in a deionized water flume (2–3°C) and washed clean; both root system samples (i.e., taproot and fine roots) were placed in a deionized ice water flume and immediately scanned (Expression 4490, Epson China Co., Ltd., Beijing, China) to acquire root system morphological images. Root length, diameter, and surface area were statistically analyzed using a WinRHIZO 2013 image analysis system (Regent Instruments Inc., Quebec City, PQ). Thereafter, all scanned root system and aboveground portion samples were dried at 65°C until a constant weight was reached as confirmed by weighing with a balance (to the nearest 0.001 g). Specific root length and surface area were calculated from the ratio of root length of the corresponding root system to biomass and that of root surface area to biomass, respectively.

The above biomass ratios of various plant organs were calculated in grams, representing dry material masses of new leaves, old leaves, stem, fine root, and coarse root of *B. ischaemum*.

#### Determination of stable carbon isotope ratio and calculation of carbon isotope discrimination

The dry material of every organ of *B. ischaemum* was ground in a mortar and passed through an 80-mesh sieve for later use; the soil sample was passed through a 0.149-mm sieve for later use. A 0.005–0.006 g of plant sample was taken and burned in the solid combustion chamber of a MultiN/C3100 total carbon/total nitrogen analyzer (Analytik Jena AG, Germany) at 1050°C sufficiently to generate CO_2_ (while recording sample total carbon content value in g kg^−1^); the CO_2_ was then introduced into a CCIA-36d-EPCO_2_ isotope mass spectrometer (Los Gatos Research, San Jose, CA) to detect δ^13^C value of sample natural abundance. δ^13^C values of plant and soil were determined with PeeDee Belemnite (PDB) as the standard using the following formula (Farquhar et al., 1982):

$$\delta^{13}C=\frac{{(}^{\text{13}}C{/}^{12}C)Sample-{(}^{\text{13}}C{/}^{12}C)PDB}{{(}^{\text{13}}C{/}^{12}C)PDB}$$

where (^13^C/^12^C)PDB is ^13^C/^12^C of PDB standard, and δ^13^C denotes the per millage of deviation between ^13^C/^12^C of the sample and that of standard sample.

Carbon isotope discrimination Δ^13^C was calculated using the following formula:

$$\Delta^{13}C(‰)=\frac{\delta^{13}C_{a}-\delta^{13}C_{p}}{1+\delta^{13}C_{p}}$$

where δ^13^C_*a*_ denotes carbon isotope ratio of CO_2_ in air, assumed to be −8%0 (Farquhar et al., 1989; Voltas et al., 2006); δ^13^C_*p*_ denotes carbon isotope ratio of CO_2_ in plant or soil sample.

### Evaluation and overall ranking of drought resistance of various indicators

For root system morphological characteristic indicators (root length, root surface area, specific root length, and specific root surface area), physiological characteristic indicators (WUE, δ^13^C, and Δ^13^C), and growth characteristic indicators (biomass and biomass ratio of each organ) of *B. ischaemum* under different moisture conditions in this study, drought resistance was judged by means of membership function values. The calculation formula of membership function (Equation 1) is

If an indicator is positively correlated with drought resistance:

$$\mu (X_{i})=\frac{X_{i}-X_{\text{min}}}{X_{\text{max}}-X_{\text{min}}},i=1,2,3......n$$

If an indicator is negatively correlated with drought resistance:

(1) $$\mu (X_{i})=1-\frac{X_{i}-X_{\text{min}}}{X_{\text{max}}-X_{\text{min}}},i=1,2,3......n$$

where μ(*X*_*i*_) is the membership function value, *X*_i_ is the measured value of an indicator, and *X*_max_ and *X*_min_ are the maximum and minimum of an indicator of all varieties tested.

For the above anti-drought physiological, ecological, and morphological indicators of *B. ischaemum*, principal component analysis (PCA) was used to extract a common factor with cumulative variance contribution rate ≥80%; common factor scores of different drought resistance indicators were then calculated and with the variance contribution rate of the principal component as the weight, weighted summations of the extracted scores were carried out to obtain the coefficient of each indicator in a composite drought resistance score model. Finally, the coefficients of the composite score model were normalized to obtain the factor weight ω(*X*_i_) of each indicator. Based on factor weight ω(*X*_*i*_) and membership function value μ(*X*_i_), the drought resistance measure *D*(*X*_*i*_) of each indicator was calculated using the following formula (Equation 2):

(2) $$D(X_{i})=\omega (X_{i})\times \mu (X_{i}),i=1,2,3......n$$

### Data analysis method

All test data were plotted using SigmaPlot10.0 (IBM Corporation, Chicago, IL, USA) and processed using SPSS21.0 (IBM Corporation, Chicago, IL, USA) statistical analysis software. Correlation analysis was conducted on transient WUE, root system morphological characteristics (root length, root surface area, specific root length, and specific root surface area), organ biomass ratio, and stable carbon isotope characteristics. Significance was determined for the above indicators, and one-way ANOVA and Duncan method were used to compare significance level of the differences between the above indicators and ratios (α = 0.05). The principal component analysis was used to extract a common factor with cumulative variance contribution rate and conducted on the weight of different drought resistance indicators.

## Results

### Photosynthetic characteristics, transient WUE, and stable carbon isotope characteristics of *B. ischaemum* under drought stress

All photosynthetic characteristic parameters decreased with decreasing soil moisture, among which net photosynthetic rate (*Pn*), transpiration rate (*Tr*), and stomatal conductance (*Gs*) under sufficient water supply (CK) were significantly higher than those under the MS and SS moisture stress conditions (Table 1). The WUEs under MS were 2.3 μmol mol^−1^, significantly higher than those under SS (2.0 μmol mol^−1^) but not significantly different from those under CK treatment (2.2 μmol mol^−1^). Although both *Pn* and *Tr* under MS treatment were significantly lower than those under CK, the stomatal conductance (*Gs*) under MS was only 0.073 mmol m^−2^ s^−1^, suggesting that the plant stomata closed under MS, so photosynthetic rate declined; however, the stomatal closure also lowered *Tr*, and eventually WUEs under MS were significantly higher than those under CK treatment. Under SS conditions, *Pn* of *B. ischaemum* declined, suggesting lower WUEs.

**Table 1:** **Changes in *B. ischaemum* gas exchange parameters under different moisture conditions**.

**Soil water treatment**	**Control (CK)**	**Mild water stress (MS)**	**Serious water stress (SS)**
Net photosynthetic rate (μmol·m^−2^ s^−1^)	8.8 ± 0.6a	7.0 ± 0.4b	5.9 ± 0.4 b
Transpiration rate (mmol m^−2^ s^−1^)	3.9 ± 0.2a	2.8 ± 0.1b	3.1 ± 0.2b
Stomatal conductance (mmol m^−2^ s^−1^)	0.1 ± 0.0a	0.1 ± 0.0 b	0.1 ± 0.0b
Water use efficiency (μmol mol^−1^)	2.2 ± 0.1ab	2.3 ± 0.1a	2.0 ± 0.1b

*CK, Control, 80% FC; MS, Mild water stress, 60% FC; SS, Serious water stress, 40% FC; WUE, water use efficiency. Daily mean ± standard error, n = 9. Different lowercase letters in the same column represent significant difference at 0.05 level among different soil water treatments*.

The changes in δ^13^C and isotope discrimination of each organ of *B. ischaemum* under drought stress are summarized in **Figure 3**. As drought stress was exacerbated, δ^13^C of each organ exhibited a rising trend. The δ^13^C values of new leaves (δ^13^C_NL_) under MS and SS treatment were −11.8 and −11.9%0, respectively, which were significantly greater than those under CK treatment. The δ^13^C values of the stem (δ^13^C_*S*_) under CK treatment was the lowest, which was −14.3%0, 1.6%0lower than SS treatment. The δ^13^C values of the stem under MS treatment was −13.6%0, which was significantly higher than SS treatment (*P* < 0.05). From the perspective of δ^13^C values of various organs, the δ^13^C values of fine root under SS treatment was the lowest, which was −11.7%0. While δ^13^C_*OL*_ was the highest, the δ^13^C values of organs under SS treatment showed no significant difference. The δ^13^C means of organs under MS treatment were manifested as follows (where δ^13^C_FR_ is δ^13^C of fine roots, δ^13^C_OL_ is δ^13^C of old leaves, and δ^13^C_CR_ is δ^13^C of coarse roots): δ^13^C_FR_ > δ^13^C_NL_ > δ^13^C_OL_ > δ^13^C_CR_ > δ^13^C_S_. In contrast, δ^13^C means of organs under CK treatment were significantly different between δ^13^C_FR_, δ^13^C_NL_, and δ^13^C_S_.

Δ^13^C values of *B. ischaemum* under different moisture treatment conditions declined with increasing drought stress. The Δ^13^C values of new leaves under CK treatment was 4.8%0, which was significantly higher than MS and SS treatments, respectively. Upon comparing Δ^13^C values of organs under the various treatment regimens, we found that Δ^13^C values generally showed the following trend: Δ^13^C_FR_ < Δ^13^C_CR_ < Δ^13^C_OL_ < Δ^13^C_S_. Δ^13^C values of organs under SS treatment were significantly different between regimens, and significant difference existed in Δ^13^C_R_ (Δ^13^C_FR_ and Δ^13^C_CR_) vs. Δ^13^C_NL_ and Δ^13^C_R_ vs. Δ^13^C_S_ for root system under CK and MS treatment. However, the difference between Δ^13^C_NL_ and Δ^13^C_S_ was insignificant.

### Effects of drought stress on biomass and root system morphology of *B. ischaemum*

Total root length and total root surface area of *B. ischaemum* under SS treatment increased significantly; total root length was 2.86 times that under CK and 2.60 times that under MS, and total root surface area was about 2.7–3.0 times those under CK and MS treatments (Table 2). Comparison of specific root length and specific root surface area of *B. ischaemum* under the three moisture treatment gradients shows that specific root length under MS treatment was significantly greater than specific root lengths under the two other treatment gradients, but specific root surface area showed no significant difference.

**Table 2:** **Changes in *B. ischaemum* root morphology parameters under different moisture conditions**.

**Soil water treatment**	**Root length (cm)**	**Root area (cm^2^)**	**Specific root length m/g**	**Specific root area m^2^/g**
CK	93.5 ± 15.4 b	8.0 ± 1.2b	253.2 ± 48.1b	0.2 ± 0.1a
MS	102.5 ± 14.3 b	7.4 ± 0.9b	421.0 ± 28.5a	0.3 ± 0.0a
SS	266.3 ± 36.0 a	22.1 ± 3.1a	264.7 ± 27.8b	0.2 ± 0.0a

*Daily mean ± standard error, n = 9. Lowercase letters indicate significant difference between different moisture treatments at the 0.05 level*.

**Figure 1:**
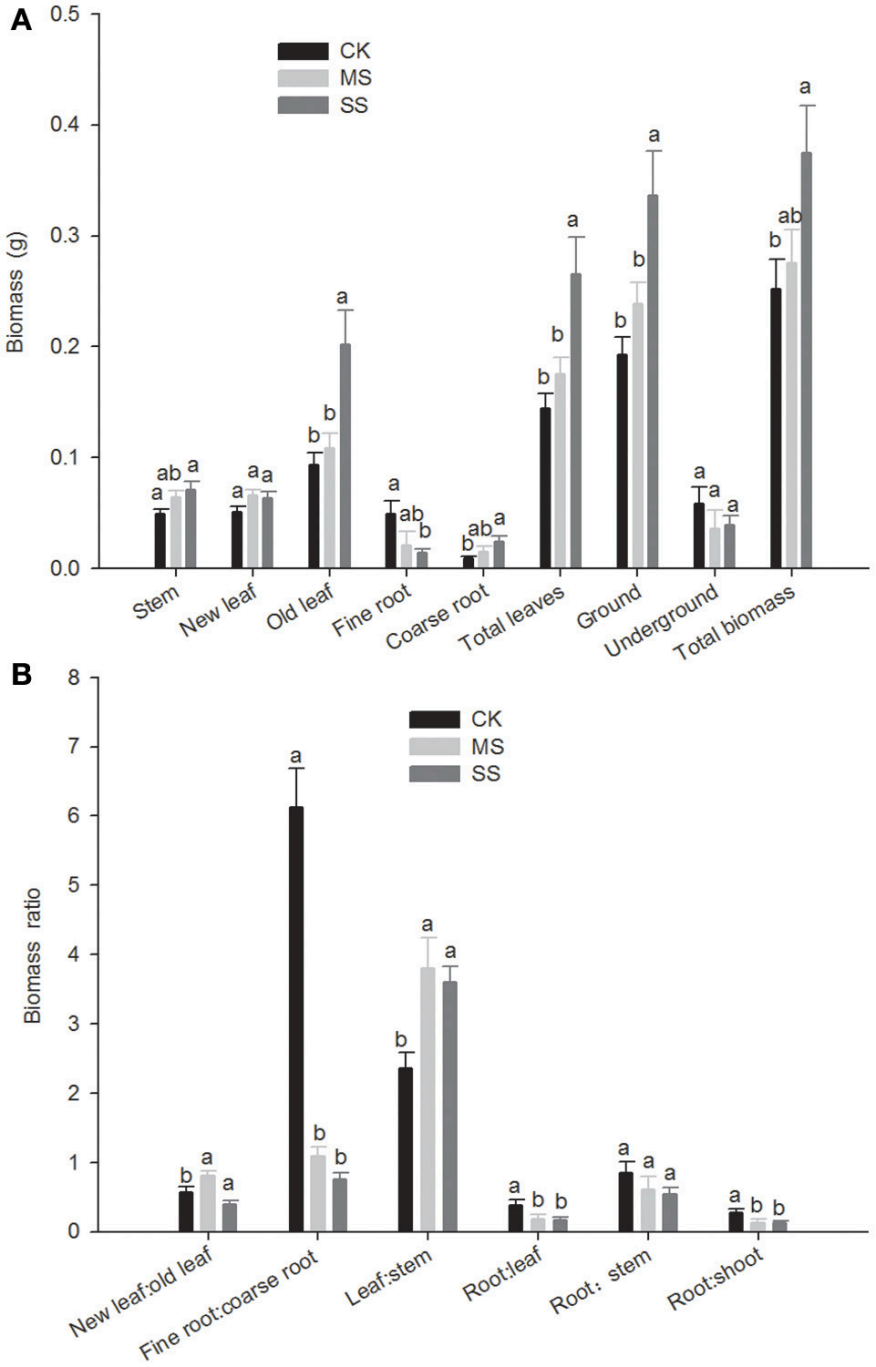
**The biomass and ratio of different parts under different water conditions *Bothriochloa ischaemum* biomass (A)** the biomass of different parts, **(B)** the biomass ratios of different parts. CK (adequate moisture), black bars with white crosshatching; MS (moderate water stress), white with black diagonal lines; SS, (severe water stress), white with black dots. Lowercase letters above each bar indicate significant difference (*P* < 0.05); vertical lines, mean ± SE.

Total biomass of *B. ischaemum* declined significantly with decreasing soil moisture, and its influence on the aboveground portion was significantly greater than that on the underground portion (Figure 1A). As to the aboveground portion, drought stress had no significant impact on new leaf biomass, but the old leaf biomass of *B. ischaemum* decreased significantly, and stem biomass under SS treatment was 0.049 g, significantly lower than that under CK treatment (0.071 g). As to the underground portion, although overall root system biomass did not differ significantly under the three moisture treatment gradients, further discrimination of the response to soil moisture between the taproot (coarse root) and lateral root (fine root) showed totally different manifestations. The coarse-root biomass was 0.024 g under sufficient moisture (CK), greater than the fine-root biomass (i.e., 0.014 g), and as soil moisture gradually decreased, the coarse-root biomass decreased while the fine root biomass increased. Under SS moisture condition, the coarse-root biomass was only 0.009 g, and the fine-root biomass was 0.049 g, 5.444 times the coarse-root biomass.

The root-to-leaf ratio under SS treatment was 0.386, significantly greater than those under the other two treatment conditions; the ratio of fine roots to coarse roots was 6.123 under SS treatment, 5.649 times that under MS treatment, and 8.056 times under CK treatment (Figure 1B). The leaf-to-stem ratio under SS treatment decreased significantly, and consequentially the root-to-shoot ratio under SS treatment was significantly greater than the same ratios under the other treatment conditions; in other words, the significant rise in root-to-shoot ratio under SS treatment was due to synergy of significantly increased fine roots and significantly decreased leaves.

### Correlation among stable carbon isotope characteristics, WUE, biomass, and root morphological characteristics of *B. ischaemum*

Correlation analysis of stable carbon isotope values, discriminations, and transient WUEs of various organs (new leaves, old leaves, stem, fine root, and coarse root) of *B. ischaemum* showed that δ^13^C and Δ^13^C of each organ exhibited very significant negative correlation (all correlation coefficients exceeded −0.989; Table 3). δ^13^C_NL_, δ^13^C_*OL*_, and δ^13^C_*S*_ exhibited significant positive correlation with WUE; Δ^13^C_*NL*_, Δ^13^C_OL_, Δ^13^C_*S*_, and Δ^13^C_FR_ exhibited significant negative correlation (*P* < 0.05) with WUE, in which the correlation coefficient (0.614) between δ^13^C_NL_ of new leaves and WUE was the maximum, followed by that of δ^13^C_OL_ (0.460), whereas the negative correlation coefficient between Δ^13^C_NL_ of new leaves and WUE was the maximum (i.e., −0.531).

**Table 3:** **Correlation analysis for carbon isotope ratio (δ^13^C), carbon isotope discrimination (Δ^13^C), and water use efficiency (WUE) in *B. ischaemum* organs**.

**δ^13^C and Δ^13^C**	**WUE**	**Δ^13^C_NL_**	**Δ^13^C_OL_**	**Δ^13^C_S_**	**Δ^13^C_FR_**	**Δ^13^C_CR_**
δ^13^C_NL_	0.61^**^	−0.99^**^	−0.58^**^	−0.35	0.31	−0.08
δ^13^C_OL_	0.46^*^	−0.55^**^	−0.99^**^	−0.15	0.25	−0.30
δ^13^C_S_	0.41^*^	−0.32	−0.11	−0.99^**^	−0.38	0.28
δ^13^C_FR_	0.37	0.48^**^	0.27	−0.33	−0.99^**^	0.64^**^
δ^13^C_CR_	−0.31	−0.13	−0.31	0.28	0.74^**^	−0.99^**^
Δ^13^C_NL_	−0.53^**^		0.53^**^	0.36	−0.39	0.13
Δ^13^C_OL_	−0.40^*^			0.11	−0.28	0.31
Δ^13^C_S_	−0.40^*^				0.35	−0.26
Δ^13^C_FR_	−0.45^*^					−0.71^**^
Δ^13^C_CR_	0.27					

*NL, New leaves; OL, old leaves; S, stem; FR, fine roots; CR, coarse roots*.

* Significant at P < 0.05;

** *Significant at P < 0.01*.

Correlation analysis of *B. ischaemum* biomass, root system morphological characteristics, and WUE (Table 4) showed that, disregarding the effects of soil moisture, WUE was very significantly correlated with root-to-shoot ratio, ratio of fine roots to coarse roots, root-to-leaf ratio, and root-to-stem ratio, and was the most correlated with the root-to-shoot ratio. Regarding the drought stress condition, the fine-root to coarse-root biomass ratio under CK treatment was the most correlated with WUE; under MS and SS treatment, the correlation between root system morphological characteristics and WUE was greater than that between biomass and WUE, and root length and WUE exhibited very significant correlation.

**Table 4:** **Correlation analysis for root morphology, biomass accumulation, and WUE in *B. ischaemum***.

**Root morphology, biomass b**		**WUE in different soil water treatments**			
		**CK (80%FC)**	**MS (60%FC)**	**SS (40%FC)**	**Total**
Biomass	RSR	−0.28	0.05	−0.30	−0.57^**^
	NL/OL	0.41	−0.22	−0.29	0.02
	FR/CR	0.61^*^	0.14	−0.20	−0.51^**^
	R/S	−0.33	0.07	−0.43	0.31^*^
	R/L	−0.26	0.08	−0.29	−0.56^**^
	R/S	−0.33	0.07	−0.50^*^	−0.50^**^
Root traits	RL	0.27	0.70^**^	0.72^**^	−0.25
	RA	0.21	0.53^*^	0.57^*^	−0.33^*^
	SRL	0.35	0.58^*^	0.25	0.51^**^
	SRA	0.29	0.30	0.54^**^	0.39^**^

*FC, Field capacity; RSR, root-to-shoot ratio; NL/OL, new leaves-to-old leaves ratio; FR/CR, fine root-to-coarse root ratio; L/ S, leaves-to-stem ratio; R/ L, root-to-leaves ratio; R/S, root-to-stem ratio; RL, root length; RA, root area; SRL, specific root length; SRA, specific root area; WUE, water use efficiency*.

* Significant at P < 0.05;

** *Significant at P < 0.01*.

Correlation analyses of stable carbon isotope characteristics of each organ of *B. ischaemum* versus biomass and root system eigenvalue, respectively, revealed that the biomass of *B. ischaemum* was significantly correlated with the stable carbon isotope characteristic of new leaves only (*P* < 0.05; *n* = 9), in which the root-to-shoot and root-to-leaf ratios exhibited significant negative correlation with δ^13^C_NL_ (Table 5). Each of the root system morphological indicators exhibited significant negative correlation with δ^13^C_S_ and significant positive correlation with Δ^13^C_S_. Moreover, total root length and total root surface area were significantly correlated with Δ^13^C_FR_ (*P* < 0.05; *n* = 9), whereas specific root surface area exhibited significant negative correlation with δ^13^C_OL_ and significant positive correlation with Δ^13^C_OL_, respectively.

**Table 5:** **Correlation analysis for carbon isotope ratio (δ^13^C), carbon isotope discrimination (Δ^13^C), Biomass and Root traits in *B. ischaemum***.

	**Biomass**						**Root traits**			
	**RSR**	**NL/OL**	**FR/CR**	**R/S**	**R/L**	**R/S**	**RL**	**RA**	**SRL**	**SRA**
δ^13^C_NL_	−0.61^**^	0.04^*^	0.02^*^	0.05^*^	−0.62^**^	0.02^*^	−0.26	−0.45^*^	0.23	−0.20
δ^13^C_OL_	−0.33	0.45	0.19	0.34	−0.33	0.07	−0.10	−0.39	−0.03	−0.46^*^
δ^13^C_S_	−0.37	0.25	0.15	0.49	−0.38	0.08	−0.57^**^	−0.46^*^	−0.55^**^	−0.59^**^
δ^13^C_FR_	−0.17	0.30	0.45	0.34	−0.15	0.21	−0.51^*^	−0.37	−0.07	−0.14
δ^13^C_CR_	0.19	0.23	0.36	0.44	0.18	0.16	0.22	0.16	−0.10	−0.11
Δ^13^C_NL_	0.54^**^	0.05^*^	0.03^*^	0.06	0.56^**^	0.04^*^	0.21	0.38	−0.20	0.17
Δ^13^C_OL_	0.29	0.50	0.21	0.42	0.29	0.08	0.03	0.32	0.04	0.43^*^
Δ^13^C_S_	0.38	0.27	0.13	0.48	0.39	0.07	0.56^**^	0.44^*^	0.55^**^	0.58^**^
Δ^13^C_FR_	0.21	0.45	0.36	0.44	0.20	0.19	0.54^*^	0.40^*^	−0.02	0.10
Δ^13^C_*CR*_	−0.17	0.25	0.39	0.48	−0.16	0.17	−0.18	−0.12	0.07	0.11

*RSR, Root-to-shoot ratio; NL/OL, new leaves-to-old leaves ratio; FR/CR, fine root-to-coarse root ratio; R/ L, root-to-leaves ratio; R/S, root-to-stem ratio; RL, root length; RA, root area; SRL, specific root length; SRA, specific root area; WUE, water use efficiency*.

* Significant at P < 0.05;

** *Significant at P < 0.01*.

### Principal component analysis of growth, morphological, and physiological characteristic indicators of *B. ischaemum*

Principal component analysis was carried out over a total of 21 indicators of *B. ischaemum* under three moisture treatment conditions, including growth indicators (biomass and ratios), physiological indicators (WUE, carbon isotope value δ^13^C, and carbon isotope discrimination Δ^13^C), and morphological indicators (root morphology), to obtain eigenvectors, factor loading, and contribution rates (Table 6). The results show that, for the first two factors out of CK and MS factor eigenvalues, their cumulative contribution rates had reached 80%, and their characteristic roots (λ) were >4.71 and >3.50, respectively; the first two factors were extracted, and variables of the same nature were classified as one type, so as to get two new composite indicators independent of each other (i.e., common factors, denoted by F1 and F2, respectively). Under SS treatment, three common factors were extracted, denoted by F1, F2, and F3, respectively. From absolute load factors of common factor loading matrix, it can be seen that, in the case of CK treatment, F1 had higher loading on root length and root surface area and F2 had higher loading on fine-root to coarse-root biomass ratio and root-to-leaf biomass ratio. In the case of MS treatment, F1 had higher loading on stable isotope ratios and isotope discriminations of new leaves and fine root, WUE, and root length; F2 had higher loading on root-to-shoot ratio and root-to-leaf ratio. In the case of SS treatment, F1 had higher loading on WUE and on stable isotope ratios and isotope discriminations of new leaves and old leaves; F2 had higher loading on leaf-to-stem ratio, stable isotope ratio, and isotope discrimination of stem; F3 had higher loading on mass ratio of new leaves to old leaves. Based on common-factor eigenvectors and contribution rates acquired, a composite score was calculated; thereby we determined the weight coefficient ω of each factor, which was used to weight a membership function value.

**Table 6:** **Changes in *B. ischaemum* principal component factor load matrix, characteristic root, synthesis score, and factor weight under different moisture conditions**.

**Factor**	**Control (CK)**				**Mild water stress (MS)**				**Serious water stress (SS)**				
	**Factor pattern**		**Synthesis score**	**Factor weight**	**Factor pattern**		**Synthesis score**	**Factor weight**	**Factor pattern**			**Synthesis score**	**Factor weight**
	**F1**	**F2**			**F1**	**F2**			**F1**	**F2**	**F3**		
RSR	0.74	0.65	2.25	0.38	0.47	−0.85	1.16	0.08	0.62	0.43	0.54	1.53	0.13
NL/OL	−0.44	0.21	−0.96	−0.16	0.58	0.49	1.98	0.14	0.63	0.26	0.69	1.48	0.13
FR/CR	0.04	−0.77	−0.38	−0.06	−0.43	0.65	−1.09	−0.08	0.25	0.64	−0.50	0.69	0.06
R/S	0.72	−0.49	1.50	0.26	0.42	0.40	1.46	0.10	0.37	0.84	0.06	1.20	0.10
R/L	0.71	0.68	2.18	0.37	0.34	−0.91	0.73	0.05	0.61	0.49	0.45	1.53	0.13
R/S	0.85	0.51	2.43	0.41	0.71	−0.59	1.98	0.14	0.68	0.60	0.39	1.70	0.15
RL	0.55	0.52	1.70	0.29	−0.98	−0.09	−3.08	−0.22	−0.98	0.03	0.17	−1.64	−0.14
RA	−0.99	0.11	−2.40	−0.41	0.98	0.00	3.06	0.22	0.79	0.33	−0.28	1.50	0.13
SRL	−1.00	0.07	−2.44	−0.41	0.90	0.34	2.93	0.21	0.74	0.02	−0.67	1.09	0.10
SRA	0.73	0.41	2.06	0.35	0.96	0.06	3.00	0.21	0.38	0.64	0.50	1.22	0.11
RSR	−0.17	0.62	−0.04	−0.01	0.52	0.78	1.89	0.14	0.76	0.05	−0.65	1.15	0.10
δ^13^C_NL_	−0.92	0.27	−2.14	−0.36	0.99	0.07	3.08	0.22	−0.98	−0.04	0.02	−1.73	−0.15
δ^13^C_OL_	−0.77	0.45	−1.65	−0.28	0.93	0.02	2.90	0.21	−0.96	0.22	0.11	−1.49	−0.13
δ^13^C_S_	−0.74	0.44	−1.58	−0.27	−0.96	0.10	−2.96	−0.21	−0.60	0.77	0.08	−0.53	−0.05
δ^13^C_FR_	0.89	−0.24	2.07	0.35	−0.99	−0.06	−3.11	−0.22	−0.75	0.58	−0.19	−0.99	−0.09
δ^13^C_CR_	0.74	0.66	2.24	0.38	0.93	0.03	2.91	0.21	−0.03	0.66	−0.54	0.19	0.02
Δ^13^C_NL_	0.91	−0.28	2.11	0.36	−0.99	−0.02	−3.07	−0.22	0.98	0.02	−0.03	1.71	0.15
Δ^13^C_OL_	0.77	−0.44	1.65	0.28	−0.92	0.02	−2.86	−0.20	0.96	−0.18	−0.11	1.53	0.13
Δ^13^C_S_	0.76	−0.40	1.65	0.28	0.96	−0.12	2.94	0.21	0.61	−0.76	−0.07	0.55	0.05
Δ^13^C_FR_	−0.90	0.19	−2.13	−0.36	0.99	0.10	3.11	0.22	0.75	−0.57	0.20	1.00	0.09
Δ^13^C_CR_	−0.73	−0.66	−2.22	−0.38	−0.95	−0.03	−2.95	−0.21	0.03	−0.66	0.55	−0.19	−0.02
Characteristic root	12.02	4.71			14.74	3.50			10.25	5.25	3.31		
Contribution rate (%)	57.24	23.42			70.20	16.66			48.81	25.00	15.76		
Cumulative contribution rate (%)	57.24	80.66			70.20	86.86			48.81	73.81	89.56		

*RSR, Root-to-shoot ratio; NL/OL, new leaves-to-old leaves ratio; FR/CR, fine root-to-coarse root ratio; R/ L, root-to-leaf ratio; R/S, root-to-stem ratio; RL, root length; RA, root area; SRL, specific root length; SRA, specific root area; δ^13^C, carbon isotope ratio; Δ^13^C, carbon isotope discrimination*.

### Membership function analysis and drought resistance evaluation of *B. ischaemum* drought indicators

A fuzzy membership function approach was applied to the above drought resistance indicators. Membership function value μ of each factor was calculated using Equation 1, to which a corresponding weight coefficient ω was assigned. A weighted membership function value was calculated using Equation 2 and served as composite drought resistance measure *D*, thereby allowing more accurate evaluation of drought resistance indicators; the greater the *D*-value, the stronger the drought resistance (Table 7). Depending on the *D*-value, drought resistance varied with soil moisture. In the case of CK treatment, the first three indicators capable of expressing drought resistance of *B. ischaemum* were root-to-stem ratio, stable isotope ratio of coarse roots (δ^13^C_CR_), and specific root length, respectively; in the case of MS treatment the first three indicators were isotope ratio of new leaves (δ^13^C_NL_), isotope ratio of coarse root (δ^13^C_CR_), and root length, respectively; as to SS treatment, the first three indicators were isotope discrimination of new leaves (Δ^13^C_NL_), mass ratio of new leaves to old leaves, and root-to-shoot ratio, respectively. This also suggests that there is an optimal indicator expressing drought resistance of *B. ischaemum*, and the optimal indicator varies with moisture treatment.

**Table 7:** **Changes in *B. ischaemum* membership function analysis and performance evaluation of drought resistance indexes under different moisture conditions**.

**Indexes**	**Mean values of subordinate function**			**Subordinate function values drought resistance comprehensive evaluation values (*****D*****-value)**			**Rank**		
	**CK**	**MS**	**SS**	**CK**	**MS**	**SS**	**CK**	**MS**	**SS**
RSR	0.55	0.48	0.66	0.21	0.04	0.09	4	13	3
NL/OL	0.53	0.50	0.72	−0.09	0.07	0.09	14	9	2
FR/CR	0.50	0.52	0.26	−0.03	−0.04	0.02	13	15	14
R/S	0.48	0.56	0.57	0.12	0.06	0.06	11	12	9
R/L	0.55	0.48	0.65	0.20	0.02	0.09	5	14	4
R/S	0.55	0.45	0.44	0.23	0.06	0.07	1	11	8
WUE	0.51	0.47	0.61	0.15	−0.10	−0.09	8	20	21
RL	0.43	0.51	0.53	−0.17	0.11	0.07	20	3	7
RA	0.40	0.47	0.48	−0.17	0.10	0.05	18	7	12
SRL	0.61	0.49	0.50	0.21	0.11	0.05	3	6	11
SRA	0.54	0.50	0.70	0.00	0.07	0.07	12	10	6
δ^13^C_NL_	0.35	0.54	0.56	−0.13	0.12	−0.08	15	1	20
δ^13^C_OL_	0.61	0.52	0.58	−0.17	0.11	−0.08	19	5	19
δ^13^C_S_	0.57	0.47	0.53	−0.15	−0.10	−0.02	16	18	17
δ^13^C_FR_	0.56	0.55	0.58	0.20	−0.12	−0.05	6	21	18
δ^13^C_CR_	0.59	0.53	0.50	0.23	0.11	0.01	2	4	15
Δ^13^C_NL_	0.48	0.47	0.68	0.17	−0.10	0.10	7	19	1
Δ^13^C_OL_	0.49	0.47	0.57	0.14	−0.10	0.08	9	16	5
Δ^13^C_S_	0.45	0.44	0.64	0.13	0.09	0.03	10	8	13
Δ^13^C_FR_	0.45	0.50	0.67	−0.16	0.11	0.06	17	2	10
Δ^13^C_CR_	0.49	0.47	0.65	−0.19	−0.10	−0.01	21	17	16

*RSR, Root-to-shoot ratio; NL/OL, new leaves-to-old leaves ratio; FR/CR, fine root-to-coarse root ratio; R/ L, root-to-leaf ratio; R/S, root-to-stem ratio; RL, root length; RA, root area; SRL, specific root length; SRA, specific root area; δ^13^C, carbon isotope ratio; Δ^13^C, carbon isotope discrimination*.

## Discussion

### Drought stress adaptation strategy of *B. ischaemum* morphological and physiological characteristics

A plant adapts to prolonged drought stress via changes in the root system morphological conformation, photosynthetic product allocation to aboveground, and underground portions, and more efficient utilization of the limited soil water. First to perceive soil moisture variation is the plant's root system, which adapts by regulating its own morphological, physiological, and biochemical characteristics. This plasticity of root system morphology in relation to its soil environment in turn influences a plant's absorption and utilization of soil moisture and nutrients and further affects growth of and biomass allocation to the aboveground portion of the plant (Sun and Zhang, 2002; Chen et al., 2012). Other scholars have proposed a “source-sink” relation in plant photosynthetic product allocation, wherein leaves are the “source” and roots are the “sink” of plant photosynthetic products; the source organ acquires the assimilation product (carbohydrate) through photosynthesis and delivers it to the sink organ via the stem, so that the individual plant grows in proportion. Such “source-sink” relationships vary with species, with CO_2_ concentration, as well as with drought stress intensity and duration (Fernández et al., 2002; Xu et al., 2005); for example, when the root system grows to reach a water source, the root and shoot compete for carbohydrates and most assimilation products are allocated to the root system, resulting in a higher root-to-shoot ratio (Xu et al., 2003; Wei et al., 2005). In this study we found that under serious drought stress, total root length, total root surface area, and root-to-shoot ratio of *B. ischaemum* increased significantly, and fine-root biomass was significantly greater than coarse-root biomass (Table 2). These results suggest that *B. ischaemum* adapts to drought stress by developing the organ (root) that allows its growth and reproduction and correspondingly reduces photosynthetic product allocation to other organs (stem and leaves), in support of the “source-sink” balance proposal. Root-to-shoot ratio increased significantly under SS treatment (Figure 1), suggesting that the plant intensifies transport of photosynthetic products from the “source” organ to the “sink” organ, thus amplifying the sink organ's share of photosynthetic product so as to adapt to the arid environment.

Marcelis and Heuvelink (2007) held that soil nutrients and moisture might change the pattern of photosynthetic product allocation by influencing organ morphological growth, and the mechanism of functional balance among organs is very complex. The current study showed that *B. ischaemum* responded to serious drought stress by allocating carbon flowing from the leaf “source” into the root system “sink” but utilized the carbon primarily to grow fine (lateral) roots and not coarse (taproot) root. A likely explanation is that the sensitivity of fine roots to nutrients and moisture is greater than that of taproot; indeed, the chief physiological function of fine roots is to absorb nutrients and moisture, whereas the taproot's main role consists of transport and support (Mei et al., 2006). Such non-uniformity of “sink” carbon allocation follows the principle of individual plant allocation optimization (Niklas, 2006); as a result, fine-root biomass increased while coarse-root biomass decreased.

**Figure 2:**
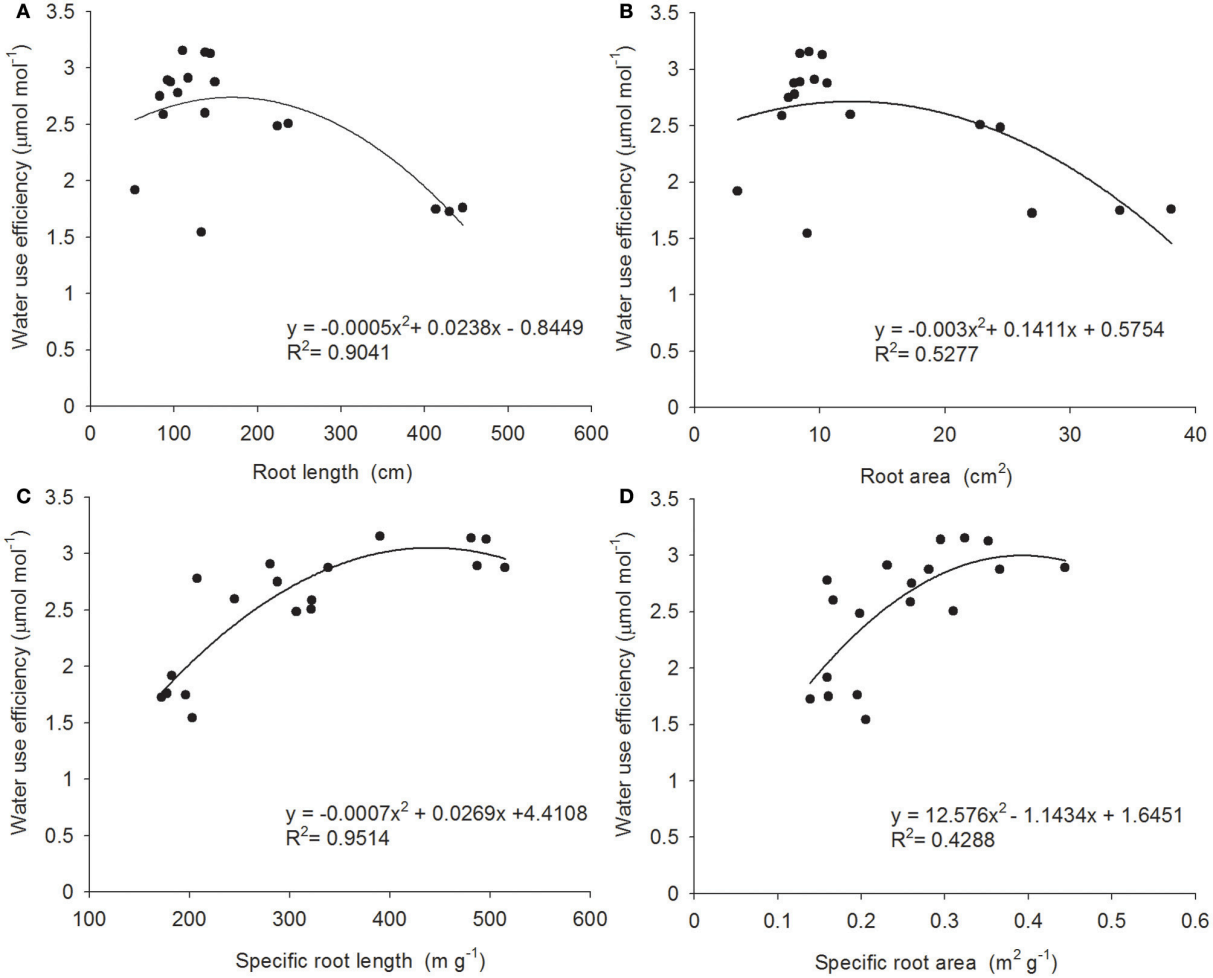
**The relation between root traits and WUE**. Relationship between four root traits and water use efficiency in *B. ischaemum*. **(A)** Root length; **(B)** root area; **(C)** specific root length; **(D)** specific root area. The line in each panel is the best- fit function of root traits and WUE.

A plant's root system also influences its WUE by varying morphological characteristics and biomass, so as to adapt to an arid environment (de Dorlodot et al., 2007; Jia and Zhang, 2008). It is commonly observed that, size, depth, and density of root system are positively correlated with drought resistance (Passioura, 1983; O'Toole and Bland, 1987; Blum and Sullivan, 1997); however, recent studies of the relationship between crop root systems and WUE show that an excessively large root system correlates negatively with aboveground yield, in that the oversized root system competes with fruit-bearing organs for photosynthetic product, consequentially leading to reduction in crop yield. This study analyzed the correlation between the morphological characteristics of the *B. ischaemum* root system and WUE; we found that all morphological characteristics of the *B. ischaemum* root system correlated significantly with WUE, and regression curves had basically similar trends and exhibited a quadratic relation (Figure 2). However, correlation levels differed and can be ranked as follows, in descending order: specific root length (*R*^2^ = 0.9514, *P* < 0.01) > total root length (*R*^2^ = 0.9041, *P* < 0.01) > total root surface area (*R*^2^ = 0.5277, *P* < 0.01) > specific root surface area (*R*^2^ = 0.4288, *P* < 0.01). Here, total root length and total root surface area had similar changing trends; the changing trend of specific root length and specific root surface area was opposite that of root length and root surface area, but each of the root system properties relative to WUE showed a peak. When a morphological indicator of root system properties exceeded the peak, WUE fell conversely; hence it is incorrect to assume that a larger and denser root system always promises better drought resistance. From the perspective of optimizing yield and moisture utilization, a plant subjected to limited water resource must have an appropriately sized root system. Furthermore, under serious drought stress, the correlation between WUE and root system morphological characteristics (i.e., root length and surface area, specific length, and specific surface area) of *B. ischaemum* was better than that between WUE and biomass, also indicating that, compared with root system biomass alone, root system morphological conformation and contribution rates of biomass allocated to fine root and coarse root appear to make more sense for maintenance of a high WUE.

### Correlation of *B. ischaemum* drought resistance indicators and screening of drought resistance expression

First, as to WUE and stable carbon isotope characteristics of *B. ischaemum*, WUE is expressed here as a ratio of the photosynthetic rate of leaves to its transpiration rate. To make full use of moisture, a plant often responds to exacerbated drought stress by closing its stomata, reducing stomatal conductance, and lowering transpiration rate; the ensuing decline in intercellular CO_2_ concentration results in less availability of CO_2_ for selective absorption by the plant, with the consequence that the δ^13^C value increases and Δ^13^C value decreases (Chaves et al., 2002; Wingate et al., 2010). Yao et al. (2015) found that the δ^13^C value of *Quercus liaotungensis* increases with increasing drought stress; Zhang et al. (2014) concluded from a study of *Ipomoea batatas* (sweet potatoes) that the Δ^13^C value falls with decreasing soil moisture; Chen et al. (2014) demonstrated that, with increasing water deficit under drought conditions, δ^13^C values of *Isatis indigotica* and *Semen cassia obtusifolia* increase significantly and Δ^13^C values of both plants decrease significantly. For *B. ischaemum*, which is a C_4_ plant, the relation of Δ^13^C versus WUE in photosynthesis engineering is:

(3) $$WUE=\frac{C_{a}(b_{1}+\phi b_{2}-\Delta^{13}C)}{1.6\nu (b_{1}+\phi b_{2}-a)}$$

where Δ^13^C is carbon isotope discrimination of each organ of *B. ischaemum, a* is carbon isotope discrimination value arising out of the CO_2_ diffusion process, *b*_1_ is the discrimination generated by gaseous CO_2_ through phosphoenolpyruvate carboxylase fixation, *b*_2_ is the discrimination of ribulose-bisphosphate carboxylase with respect to ^13^C, ψ denotes the proportion of CO_2_ released from a bundle sheath to mesophyll cells, *C*_*a*_ is concentration of CO_2_ in air (*C*_*i*_ is intercellular CO_2_ concentration of *B. ischaemum* leaf), ν is vapor pressure deficit (where ν = μ(1–RH), μ is vapor pressure of air, RH is relative humidity of air), and 1.6 is the conversion coefficient between stomatal conductance to air and that to CO_2_.

In theory, WUE is negatively correlated with Δ^13^C, and this study shows that the correlation coefficient between WUE of *B. ischaemum* and Δ^13^C of leaves was −0.531 (Table 3), exhibiting a highly significant negative correlation (*P* < 0.01). Meanwhile, WUE and δ^13^C of leaves exhibited a highly significant positive correlation—-a finding that is consistent with those of other studies showing that leaf Δ^13^C is more appropriately used to study a plants' relation to WUE, and is corroborated by other studies of C_4_ plants (Zhang et al., 2009; Wingate et al., 2010). Therefore, by determining δ^13^C or Δ^13^C value of C_4_ plant leaves, it is likewise feasible to judge moisture utilization of *B. ischaemum* under drought conditions.

Second, biomass usually serves as an indicator reflecting plant growth, and previous studies on growth and WUE demonstrate that WUE for a plant under an arid environment correlates positively with root-to-shoot ratio and negatively with both aboveground and total biomass (Saraswathi and Paliwal, 2011; Chen et al., 2014). We found in this study that WUE indeed correlated positively with root-to-shoot ratio under MS treatment (Table 4), in agreement with the results of previous studies; however, under SS and CK treatment, WUE of *B. ischaemum* correlated negatively with root-to-shoot ratio, reflecting the result of the quadratic curves we created of WUE vs. root system morphological characteristics under different drought stress treatment conditions; that is, root system morphological characteristics relative to WUE had a peak, and WUE would decline when root system growth became redundant. Under serious drought stress, the root-to-stem ratio of *B. ischaemum* increased significantly; this suggests that a plant improves WUE of the whole plant by reducing biomass allocation to stem and increasing root system growth, so as to adapt to the arid environment. We also found that, δ^13^C_NL_ and Δ^13^C_NL_ of *B. ischaemum* correlated very significantly with root-to-shoot ratio and root-to-leaf ratio, with respective correlation coefficients of −0.61 and 0.54 (*P* < 0.01), suggesting that it is likewise feasible to use δ^13^C_*NL*_ and Δ^13^C_NL_ to study their relations with growth indicators such as root-to-shoot and root-to-leaf ratios.

Third, unlike with use of δ^13^C_*NL*_ and Δ^13^C_NL_ to express plant WUE and biomass, δ^13^C_S_ and Δ^13^C_*S*_ of *B. ischaemum* were most correlated with root system morphology (*P* < 0.05), and only under the drought stress treatments (the MS and SS treatments) could root system morphology be significantly correlated with WUE. According to the hypothesis of “source-sink” relations in photosynthetic product allocation, during the plant germination phase the root system is the main sink, and after the plant receives illumination, leaves become the main carbon source; thereafter, the root system loses its dominant role and the stem portion becomes the main sink. During different phases of plant growth, the intensity of each sink differs, so that the allocation pattern of plant photosynthetic products also changes (Marcelis and Heuvelink, 2007). Sampling time in this study was the flowering phase of *B. ischaemum*, and during that growth phase sink intensity gradually migrated from root system to the stem; thus, it is also feasible to represent root system morphology via stable isotope value and isotope discrimination of the stem.

In general, δ^13^C_NL_ and Δ^13^C_NL_ could be used to characterize plant WUE and biomass, while ^13^C_S_ and Δ^13^C_S_ could express root system morphological characteristics. This corroborates our proposal in the introduction: The stable isotope approach can provide a quick and easy method to selectively breed plants with good growth, high WUE, and reasonably structured root system in the Loess Plateau under limited moisture conditions. However, breeders using the stable isotope approach must consider the influence on the final result of carbon fractionation among the sampling organ and various organs.

Finally, we found from the above analysis that mechanisms of plant drought resistance are complex and variable, and the influential indicators also mutually interacted, so reasonable selection of indicators is a key to drought resistance identification (Li et al., 2014). Previous researchers have used this method mostly to compare differences in drought resistance between crop varieties, between clonal varieties of the same plant, or between shrub species (Dai et al., 2016; Liu et al., 2016; Qiu et al., 2014). Based on this information, we selected 21 drought-related indicators (growth, morphology, and physiology) of *B. ischaemum* under different drought stress conditions and used the membership function approach and PCA to obtain measures of drought resistance (*D*-value). This method not only integrated relations among indicators but also considered the importance of each indicator; by comparing *D*-values, we screened for the optimal indicator that expresses drought resistance of *B. ischaemum* under different drought stress conditions.

Our study found that (Tables 6, 7), when moisture supply was sufficient, the growth, morphological, and physiological indicators most indicative of *B. ischaemum* drought resistance were root-to-stem ratio, specific root length, and δ^13^C_CR_; when moisture stress was mild, the morphological and physiological indicators most indicative of drought resistance were the root length, δ^13^C_NL_, and δ^13^C_CR_; when moisture stress was serious, the physiological and growth indicators most indicative of drought resistance were δ^13^C_NL_, root-to-shoot ratio, and ratio of new leaves to old leaves. These results only partially support our original proposal that we could identify an optimal indicator expressing drought resistance of *B. ischaemum*. Under each of the three moisture treatment conditions we found different optimal indicators expressing growth, morphology, and physiology of *B. ischaemum*; therefore, we provide here a theoretical reference for screening plant drought resistance indicators under different moisture conditions in the Loess Plateau. However, the difficulty we experienced in finding a single optimal indicator indicating plant drought resistance suggests that plant anti-drought mechanisms differ under various moisture conditions. In light of this difficulty, further in-depth study will be required to determine the specific plant and environmental characteristics that trigger the physiological, morphological, and growth changes we measured herein under varying conditions of *B. ischaemum* drought stress.

### Difference in carbon isotope composition of each organ of *B. ischaemum*

**Figure 3:**
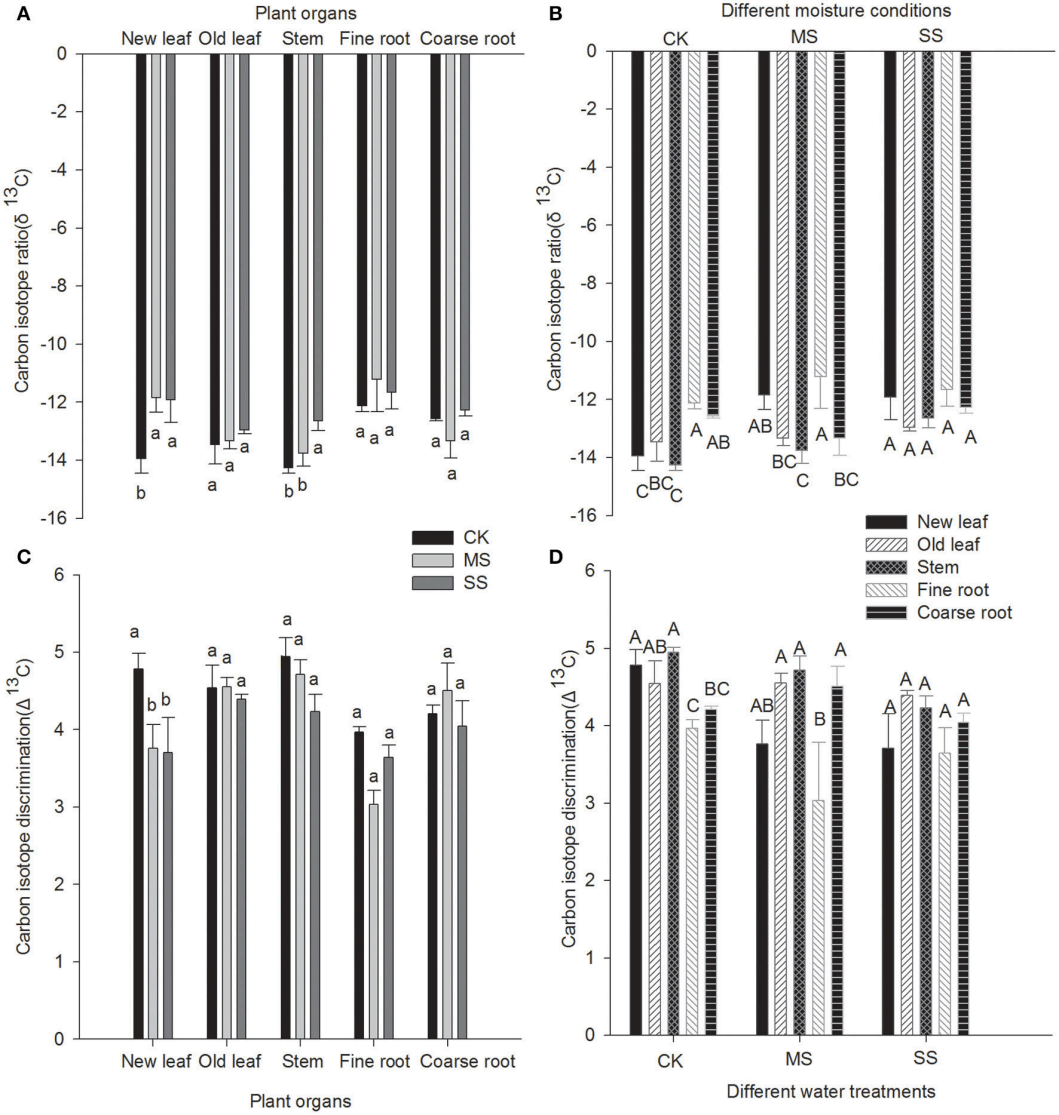
**Carbon isotope ratio and carbon isotope discrimination of various plant organs under different water treatments (A)** Mean carbon isotope ratio of various plant organs; **(B)** Mean carbon isotope ratio of different water treatments; **(C)** Mean carbon isotope discrimination of various plant organs; **(D)** Mean carbon isotope discrimination of different water treatments. Lowercase letters indicate significant difference between different water treatments at the 0.05 level. Capital letters represent significant difference among different organs at 0.05 level.

Many researchers have performed carbon isotope fractionation analysis of different organs of C_4_ plants such as corn (Zhang et al., 2009), C_3_ plants such as sweet potatoes (Zhang et al., 2014), and aerobic rice (Zhao et al., 2004), finding that δ^13^C values of all organs of C_3_ plants differed significantly, whereas δ^13^C differences between leaf and stem in C_4_ plants were insignificant; both seeds and root systems of C_3_ and C_4_ plants were more prone to ^13^C enrichment than their leaves and stems. ANOVA of the current study (Figure 3) showed that differences in δ^13^C and Δ^13^C values among organs under CK and MS conditions reached significant levels, whereas the differences among organs under SS treatment were insignificant, probably because different moisture treatment conditions influenced ^13^C fractionation of *B. ischaemum*. To summarize the differences among organs, the differences in Δ^13^C values among stem, new leaves, and old leaves were insignificant (Table 2); ^13^C fractionation did not occur between leaves and stem of *B. ischaemum*, but Δ^13^C values of both stem and leaves were significantly greater than those of root, suggesting that ^13^C fractionation had occurred between root and foliage. The root system is the “sink” organ of a plant, and its carbon undergoes the combined effects of environmental and metabolic factors during accumulation. Brugnoli and Farquhar (1998) and Leavitt and Long (1986) proposed three causes for ^13^C fractionation of a plant: First, variation in chemical composition of each organ leads to ^13^C fractionation, in that every chemical component has its own stable δ^13^C value; a higher cellulose content in an organ is more prone to ^13^C enrichment, and the higher the lignin and fat content, the lower the ^13^C content. Second, the physiological and ecological characteristics of plant organs differ extensively. Therefore, secondary fractionation of ^13^C might occur during carbohydrate synthesis; during carbohydrate export, loading, and unloading via phloem; and during transport of photosynthetic product from the “source” to the “sink.” Third, respiration characteristics of different organs differ greatly; because plant organs tend to utilize ^12^C in respiration, ^13^C is enriched in tissue. Therefore, comparisons of δ^13^C and Δ^13^C values between plants will require specific analysis of results from different organs of each individual plant.

## Author contributions

PL and YL conceived and designed the experiments. GX, LX performed the experiments. ZR and ZL analyzed the data. YL wrote the manuscript.

## Funding

This research was funded by the National Natural Science Foundation of China (No. 41471226, No. 41271290, and No. 41330858), National key research and development program (No. 2016YFC0402404) and the School foundation of Xi'an University of Technology (310-252071506).

### Conflict of interest statement

The authors declare that the research was conducted in the absence of any commercial or financial relationships that could be construed as a potential conflict of interest.

## References

[B1] Blum A. Sullivan C. Y. (1997). The effect of plant size on wheat response to agents of drought stress I. Root drying. Aust. J. Plant Physiol. 24, 35–41. 10.1071/PP96022

[B2] Brugnoli E. Farquhar G. D. (1998). Photosynthesis, in Physiology and Metabolism, ed Leegood R. C. Sharkey T. D. Caemmerer S. (New York, NY: Kluwer Academic Publishers), 399–434.

[B3] Chaves M. M. Pereira J. S. Maroco J. Rodrigues M. L. Ricardo C. P. Osório M. L.. (2002). How plants cope with water stress in the field? Photosynthesis and growth. Ann. Bot. 89, 907–916. 10.1093/aob/mcf105 12102516 PMC4233809

[B4] Chen L. Wang S. F. Liu R. L. Wang H. (2012). Changes of root morphology and rhizosphere processes of wheat under different phosphate supply. Plant Nutr. Fertil. Sci. 2, 324–331.

[B5] Chen P. Meng P. Zhang J. S. He C. X. Jia C. R. Li J. Z. (2014). Effects of drought stress on growth and water use efficiency of two medicinal plants. Chin. J. Appl. Ecol. 5, 1300–1306. 10.13287/j.1001-9332.2014.0028 25129928

[B6] Chen T. Feng H. Y. Xu S. J. Qiang W. Y. An L. Z. (2013). Stable carbon isotope composition of desert plant leaves and water-use efficiency. J. Desert Res. 22, 288–291. 10.3321/j.issn:1000-694X.2002.03.016

[B7] Cheng J. Hu T. M. Cheng J. M. (2010). Responses of distribution of *Bothriochloa ischaemum* community to hydrothermal gradient in loess plateau. Acta Agrectir Sin. 2, 167–171. 10.11733/j.issn.1007-0435.2010.02.005

[B8] Dai X. D. Xu X. Z. Zhu C. C. Yang Y. F. Wang C. Y. Yang X. P. (2016). Seeding stage response to different water availability and drought resistance evaluation of foxtail millet. Crops 1, 140–143. 10.16035/j.issn.1001-7283.2016.01.026

[B9] de Dorlodot S. Forster B. Pagès L. Price A. Tuberosa R. Draye X. (2007). Root system architecture: opportunities and constraints for genetic improvement of crops. Trends Plant Sci. 10, 474–481. 10.1016/j.tplants.2007.08.012 17822944

[B10] FAO-UNESCO (1977). Soil Map of the World: 1:5 000 000. Paris: UNESCO.

[B11] Farquhar G. D. Ehieringer J. R. Hibick K. T. (1989). Carbon isotope discrimination and photosynthesis. Annu. Rev. Plant Biol. 1, 503–537. 10.1146/annurev.pp.40.060189.002443

[B12] Farquhar G. D. O'Leary M. H. Berry J. A. (1982). On the relationship between carbon isotope discrimination and the intercellular carbon dioxide concentration in leaves. Aust. J. Plant Physiol. 9, 121–137. 10.1071/PP9820121

[B13] Fernández R. J. Wang M. Reynolds J. F. (2002). Do morphological changes mediate plant responses to water stress? A steady—state experiment with two C4 grasses. New Phytol. 1, 79–88. 10.1046/j.1469-8137.2002.00438.x 33873299

[B14] Ghashghaie J. Badeck F. W. Girardin C. Huignard C. Aydinlis Z. Fonteny C. (2016). Changes and their possible causes in delta C-13 of dark-respired CO2 and its putative bulk and soluble sources during maize ontogeny. J. Exp. Bot. 67, 2603–2615. 10.1093/jxb/erw075 26970389

[B15] Huang Z. B. Cheng J. M. Zhao S. W. Xin X. G. Liu X. J. (2004). Models of rainwater harvesting system and their benefit evaluation in semi-arid areas. Trans. Chin. Soc. Agric. Eng. 2, 301–304. 10.3321/j.issn:1002-6819.2004.02.071

[B16] Jaleel C. A. Gopi R. Sankar B. Gomathinayagam M. Panneerselvam R. (2008). Differential responses in water use efficiency in two varieties of *Catharanthus roseus* under drought stress. C. R. Biol. 1, 42–47. 10.1016/j.crvi.2007.11.003 18187121

[B17] Jia W. Zhang J. (2008). Stomatal movements and long-distance signaling in plangs. Plant Signal. Behav. 10, 772–777. 10.4161/psb.3.10.6294 PMC2634372 19513229

[B18] Leavitt S. W. Long A. (1986). Stable carbon isotope variability in tree foliage and wood. Ecology 67, 1002–1010. 10.2307/1939823

[B19] Li S. J. Chen L. Peng S. D. Chen S. W. Wang Q. F. Lin L. B. (2014). Screening of drought-tolerant *Brassica napus* L. varieties and analysis on their physiologic and biochemical variations under drought stress. Agric. Sci. Tech. 4, 596–604. 10.16175/j.cnki.1009-4229.2014.04.035

[B20] Liu G. H. Chen Q. J. Wu P. Qu Y. Y. Gao W. W. Yang J. S. (2016). Screening and comprehensive evaluation of drought resistance indices of cotton at blossing and boll-forming stages. J. Plant Genet. Res. 1, 53–62. 10.13430/j.cnki.jpgr.2016.01.009

[B21] Lu Y. H. Fu B. J. Feng X. M. Zeng Y. Liu Y. Chang R. Y. (2012). A policy-driven large scale ecological restoration: quantifying ecosystem services changes in the Loess Plateau of China. PLoS ONE 2:e31782 10.1371/journal.pone.0031782 PMC3280995 22359628

[B22] Marcelis L. F. M. Heuvelink E. (2007). Concepts of modelling carbon allocation among plant organs, in Functional-Structural Plant Modelling in Crop Production, eds Vos J. Marcelis L. F. M. de Visser P. H. B. Struik P. C. Evers J. B. (Dordrecht: Springer), 103–111.

[B23] Martin B. Tauer C. G. Lin R. K. (1999). Carbon isotope discrimination as a tool to improve water-use efficiency in tomato. Crop Sci. 6, 1775–1783. 10.2135/cropsci1999.3961775x

[B24] Mei L. Wang Z. Q. Han Y. Z. Gu J. C. Wang X. R. Cheng Y. H. (2006). Distribution patterns of *Fraxinus mandshurica* root biomass, specific root length and root length density. Chin. J. Appl. Ecol. 1, 1–4. 16689223

[B25] Mu Z. X. Zhang S. Q. Hao W. F. Lang A. H. Lang Z. S. (2005). The effect of root morphological traits and spatial distribution on WUE in maize. Acta Ecol. Sin. 11, 2895–2890. 10.3321/j.issn:1000-0933.2005.11.015

[B26] Niklas K. J. A. (2006). Phyletic perspective on the allometry of plant biomass-partitioning patterns and functionally equivalent organ-categories. New Phytol. 1, 27–40. 10.1111/j.1469-8137.2006.01760.x 16771980

[B27] O'Toole J. C. Bland W. L. (1987). Genotypic variation in crop plant root systems. Adv. Agron. 41, 91–145.

[B28] Passioura J. B. (1983).Root and drought resistance. Agric. Water Manag. 7, 265–280. 10.1016/0378-3774(83)90089-6

[B29] Pu W. F. Li G. L. Zhang M. Wang D. Wang L. P. Ji Z. B. (2010). Effects of drought stress on root characteristics and physiological indexes of *Glycine soja* and *Glycine max*. Soybean Sci. 29, 615–622.

[B30] Qiu Q. Pan X. Li J. Y. Wang J. H. Dong L. Ma J. W. (2014). Comparison on biomass allocation and leaf water use efficiency and δ13C of 20 shrub seedlings in Tibetan Plateau. J. Northwest Forest. Univ. 4, 8–14. 10.3969/j.issn.1001-7461.2014.04.02

[B31] Qu C. M. Han X. G. Su B. Huang J. H. Jiang G. M. (2001). The characteristics of foliar δ13C values of plants and plant water use efficiency indicated by δ13C values in two fragmented rainforests in Xishuangbanna, Yunnan. Acta Bot. Sin. 43, 186–192. 10.3321/j.issn:1672-9072.2001.02.012

[B32] Ray I. M. Townsend M. S. Muncy C. M. (1999). Heritabilities and interrelationships of water-use efficiency and agronomic traits in irrigated alfalfa. Crop Sci. 39, 1088–1092. 10.2135/cropsci1999.0011183X003900040022x

[B33] Saraswathi S. G. Paliwal K. (2011). Drought induced changes in growth, leaf gas exchange and biomass production in *Albizia lebbeck* and *Cassia siamea* seedlings. J. Environ. Biol. 32, 173–178. 21882651

[B34] Shan L. (2002). Development trend of dryland farming technologies. Sci. Agric. Sin. 7, 848–855. 10.3321/j.issn:0578-1752.2002.07.021

[B35] Su B. Han X. G. Li L. H. Huang J. H. Bai Y. F. Qu C. M. (2000). Responses of δ13C value and water use efficiency of plant species to environmental gradients along the grassland zone of Northeast China Transect. Acta Phytoecol. Sin. 24, 648–655.

[B36] Sun H. G. Zhang F. S. (2002). Morphology of wheat roots under low-phosphorus stress. Chin. J. Appl. Ecol. 3, 295–299. 12132156

[B37] Sun X. K. Fan Z. P. Wang H. Bai J. Zhang Y. Deng D. Z. (2008). Photosynthetic characteristics and water use efficiency of three broad-leaved tree species in the horqin sandland. J. Arid Land Res. Environ. 10, 188–194. 10.13448/j.cnki.jalre.2008.10.032

[B38] Toorchi M. Shashidhar H. E. Hittalmani S. Gireesha T. M. (2002). Rice root morphology under contrasting moisture regimes and contribution of molecular marker heterozygosity. Euphytica 126, 251–257. 10.1023/A:1016317906963

[B39] Voltas J. Serrano L. Hernández M. Pemán J. (2006). Carbon isotope discrimination, gas exchange and stem growth of four Euramerican hybrid poplars under different watering regimes. New Forests 3l, 435–45l. 10.1007/s11056-005-0879-7

[B40] Wang J. Wu F. Q. Meng Q. Q. (2006). Analysis on soil moisture character of dry orchard on hilly and gully regions on the Loess Plateau. J. Northwest Forest. Univ. 5, 65–68. 10.3969/j.issn.1001-7461.2006.05.015

[B41] Wei L. L. Zhang X. Q. Hou Z. H. Xu D. Y. Yu X. B. (2005). Effects of water stress on photosynthesis and carbon allocation in Cunninghamia Lanceolata seedlings. Acta Phytoecol. Sin. 3, 394–402.

[B42] Wei S. L. Liu J. N. Wang T. Zhang W. Yu Z. L. (2004). Effects of nitrogen ion implantation on seed germination and root development in Glycyrrhiza uralensis and possible mechanism. Acta Prataculturae Sin. 5:112–115. 10.3321/j.issn:1004-5759.2004.05.020

[B43] Wingate L. Ogée J. Burlett R. Bosc A. Devaux M. Grace J. (2010). Photosynthetic carbon isotope discrimination and its relationship to the carbon isotope signals of stem, soil and ecosystem respiration. New Phytol. 2, 576–589. 10.1111/j.1469-8137.2010.03384.x 20663061

[B44] Xu B. C. Shan L. Chen F. M. (2007). Comparison of ecophysiological characteristics of seven plant species in semiarid loess hilly-gully region. Chin. J. Appl. Ecol. 990–996. 17650846

[B45] Xu B. C. Shan L. Huang J. Huang Z. B. (2003). Comparison of water wse efficiency and root shoot ratio in seedling stage of swltehgrass (*Panicum virgatum*) and old world blue stems(OotkriovMoa igchaemnm) under different soil water conditions. Acta Prataculturae Sin. 4, 73–77.

[B46] Xu L. R. Zhang J. M. Ding S. Y. (1997). Characteristic on the steppe of *Bothriochloa ischaemum* in loess plateau and its geographical significance. Acta Bot. Boreali Occidentalia Sin. 17, 88–93.

[B47] Xu W. Z. Xu B. C. Duan D. P. Niu F. R. (2010). Study on the photosynthetic characteristics of *Bothriochloa ischaemum* under different water and nutrient conditions 1.diurnal variation of photosynthesis. Acta Agrestia Sin. 5, 629–635.

[B48] Xu Z. Z. Zhou G. S. Xiao C. W. Wang Y. H. (2005). Interacive effects of doubled atmospheric CO2 concentrations and soil drought on whole plant carbon allocation in two dominant desert shrubs. Acta Phytoecol. Sin. 2, 281–288.

[B49] Yao Z. M. Chai Y. Feng B. Xu Z. Y. (2015). Differences in water use efficiency of Abies holophylla seedlings from seven provenances under water stress. J. Beijing Forest. Univ. 5, 1–8. 10.13332/j.1000-1522.20140452

[B50] Zhang C. Z. Zhang J. B. Zhao B. X. Zhang H. Huang P. Li X. P. (2009). Relationships among water use efficiency, carbon isotope discrimination, and specific leaf area in maize. Acta Agron. Sin. 6, 1115–1121. 10.3724/sp.j.1006.2009.1115

[B51] Zhang H. Zhu L. D. Ning Y. W. Zhang C. Z. Zhang Y. C. (2014). Effect of water deficit condition on water use efficiency and carbon isotope discrimination in sweet potato. Soil 5, 806–813. 10.13758/j.cnki.tr.2014.05.006

[B52] Zhang J. H. Han H. Y. Lei Y. K. Yang W. B. Li Y. H. Yang D. F. (2012). Correlations between distribution characteristics of Artemisia ordosica root system and soil moisture under different fixation stage of sand dunes. J. Southwest Forest. Univ. 6, 1–5. 10.3969/j.issn.2095-1914.2012.06.001

[B53] Zhao B. Z. Kondo M. Maeda M. Ozaki Y. Zhang J. B. (2004). Water-use efficiency and carbon isotope discrimination in two cultivars of upland rice during different developmental stages under three water regimes. Plant Soil 261, 61–75. 10.1023/B:PLSO.0000035562.79099.55

[B54] Zhou Y. C. Fan J. W. Zhong H. P. Zhang W. Y. (2013). Relationships between altitudinal gradient and plant carbon isotope composition of grassland communities on the Qinghai-Tibet Plateau, China. Sci. China Earth Sci. 56, 311–320. 10.1007/s11430-012-4498-9

[B55] Zou H. Gao G. Y. Fu B. J. (2016). The relationship between grassland ecosystem and soil water in arid and semi-arid areas: a review. Acta Ecol. Sin. 36, 3127–3136. 10.5846/stxb201506211251

